# Modelling viscoacoustic wave propagation with the lattice Boltzmann method

**DOI:** 10.1038/s41598-017-10833-w

**Published:** 2017-08-31

**Authors:** Muming Xia, Shucheng Wang, Hui Zhou, Xiaowen Shan, Hanming Chen, Qingqing Li, Qingchen Zhang

**Affiliations:** 1 State Key Laboratory of Petroleum Resources and Prospecting, CNPC Key Lab of Geophysical Exploration, China University of Petroleum, 102249 Beijing, China; 2 Beijing Aeronautical Science and Technology Research Institute of COMAC, 102211 Beijing, China; 3 South University of Science and Technology of China, Shenzhen, 518055 China

## Abstract

In this paper, the lattice Boltzmann method (LBM) is employed to simulate wave propagation in viscous media. LBM is a kind of microscopic method for modelling waves through tracking the evolution states of a large number of discrete particles. By choosing different relaxation times in LBM experiments and using spectrum ratio method, we can reveal the relationship between the quality factor Q and the parameter *τ* in LBM. A two-dimensional (2D) homogeneous model and a two-layered model are tested in the numerical experiments, and the LBM results are compared against the reference solution of the viscoacoustic equations based on the Kelvin-Voigt model calculated by finite difference method (FDM). The wavefields and amplitude spectra obtained by LBM coincide with those by FDM, which demonstrates the capability of the LBM with one relaxation time. The new scheme is relatively simple and efficient to implement compared with the traditional lattice methods. In addition, through a mass of experiments, we find that the relaxation time of LBM has a quantitative relationship with Q. Such a novel scheme offers an alternative forward modelling kernel for seismic inversion and a new model to describe the underground media.

## Introduction

Since the 1960s, diverse numerical methods, such as finite difference method (FDM)^1, 2^, finite element method (FEM)^3, 4^ and pseudo-spectral method (PSM)^5, 6^, have been applied in seismology. These methods provide direct solutions of the macroscopic continuum equations, i.e., the Navier-Stokes equations or the wave equations, therefore, the solutions are restricted by the preconditions of the macroscopic equations.

Another series of discrete methods named lattice Boltzmann methods (LBM) have been shown to be effective in modelling seismic wave propagation through tracking the moving states of the discrete particles at the microscopic level. Originated from the lattice gas automata (LGA)^7, 8^, LBM has been widely used in computational fluid dynamics (CFD)^9^.

Margolus *et al*.^10^ are the pioneers who applied LGA to simulate acoustic wave propagation in the two-dimensional (2D) homogeneous fluid medium. After that, LGA was employed to model seismic P-waves in 2D homogeneous^11^ and heterogeneous media^12^. Although LGA is unconditionally stable and has no round-off error, such a forward modelling scheme suffers from statistical noise and is hard to simulate wave propagation in complex media. McNamara and Zanetti^13^ presented an alternative technique named LBM to model the lattice gas with a Boltzmann equation, which eliminates the statistical noise in LGA.

Mora and his co-workers improved LBM by introducing phonon into the original LBM, and they called this new scheme as phononic lattice solid (PLS)^14–17^. Their work was a bold attempt and they really developed a promising theory, however, the computation was a little bit time-consuming because of the complex collision term. Fortunately, a simplified LBM equation with one relaxation time in the collision term was soon developed^18–20^, and this simplified model was referred as LBM-BGK.

As the LBM-BGK model is relatively simple to implement and has a high computational efficiency, a lot of experts from various research areas have been keeping their eyes on such a new scheme. Apart from the applications in CFD, LBM was used in the simulation of different types of waves, such as shock wave^21, 22^, acoustic wave^23–26^, aeroacoustic wave^27^, acoustic streaming^28, 29^, elastic waves^30, 31^, and so on.

In this paper, a novel scheme based on the discrete lattice Boltzmann equation with a single relaxation time is adopted to simulate viscoacoustic wave propagation. A preliminary relationship between the relaxation time (*τ*) in lattice Boltzmann equation and quality factor (Q) in viscoacoutic equation is found and verified through vast numerical experiments. We should keep in mind that Q for the Kelvin-Voigt model is frequency dependent, but the Q value shown in this paper is a constant which corresponds to the point when the reference frequency is chosen as the dominant frequency of the seismic source. The relationship between Q and relaxation time presented in this paper would help us capturing the wave propagation phenomenon as well as interpreting the attenuation of wave propagation in complex viscous media.

## Results

### Homogeneous model

**Figure 1:**
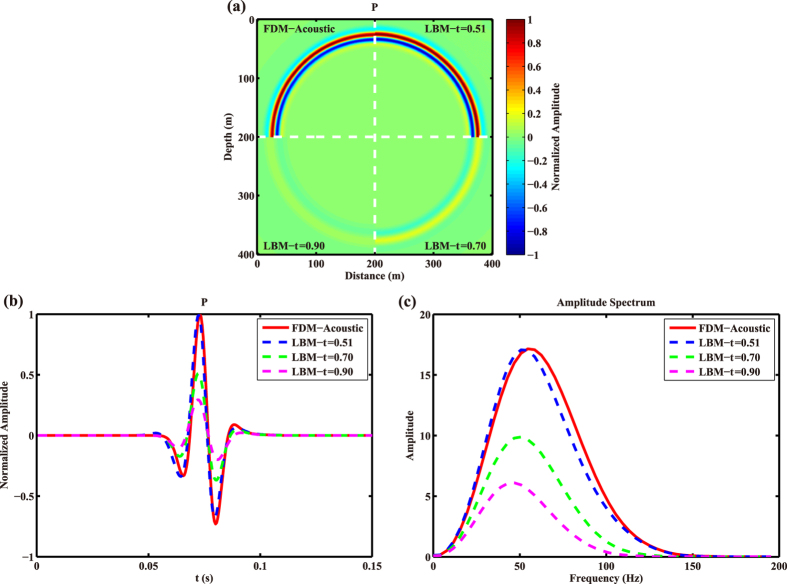
Snapshots (**a**), seismic traces (**b**) and the corresponding amplitude spectra (**c**) obtained by acoustic FDM and LBM with *τ* = 0.51, LBM with *τ* = 0.70, and LBM with *τ* = 0.90.

In numerical experiments, we use the lattice Boltzmann equation with only one relaxation time (*τ*), and *τ* is related to the viscosity of the media^30^, which means the wavefields obtained by LBM contain viscous effects. To investigate the influence of the viscosity on the wavefields, we contrast the acoustic wavefield without considering viscosity to those simulated by LBM with different relaxation times. For simplicity, we set a 2D homogeneous model with discrete grids of 401 × 401. The spatial intervals are Δ*x* = Δ*z* = 1.0 m, time interval is Δ*t* = 0.5 ms. P-wave velocity is 1,155 m/s and density is 1,000 kg/m^3^. The dynamic evolution of LBM is carried out in the dimensionless lattice space and time domain, and some of the above parameters are transformed into lattice units in our numerical experiments^30, 32^. A Ricker wavelet source^33^ with a dominant frequency of 50 Hz is imposed on the middle point of the discrete model. Three different relaxation times of 0.51, 0.70 and 0.90 are used in LBM simulations, and the acoustic wavefields calculated by FDM with similar model parameters are shown for comparison. The resultant snapshots by FDM and LBM are shown in Fig. 1a. To further depict the difference of the wavefields calculated by FDM and LBM, the seismic traces at (121 m, 121 m) are shown in Fig. 1b.

It is noticed from Fig. 1a and b that the amplitude and phase vary as the relaxation time varies. More accurately, the amplitude of the wavefield calculated by LBM declines with the rises of the relaxation time. When the relaxation time is approaching 0.5, the computed snapshot obtained by LBM is almost the same as the acoustic result by FDM; when the relaxation time is approaching 1.0, the wavefields suffer from visible attenuation, great amplitude decrease and slight phase dispersion. Such a conclusion is further confirmed by the amplitude spectra of seismic traces obtained by LBM and acoustic FDM in Fig. 1c, from which we can see the peak frequencies, at which the amplitude of the spectrum is the maximum, corresponding to the results by LBM are lower than the result by acoustic FDM. Additionally, the peak frequency and amplitude of the spectrum decrease with the increase of the relaxation time of LBM. That is to say the wavefields calculated by LBM contain viscoacoustic effects.

**Figure 2:**
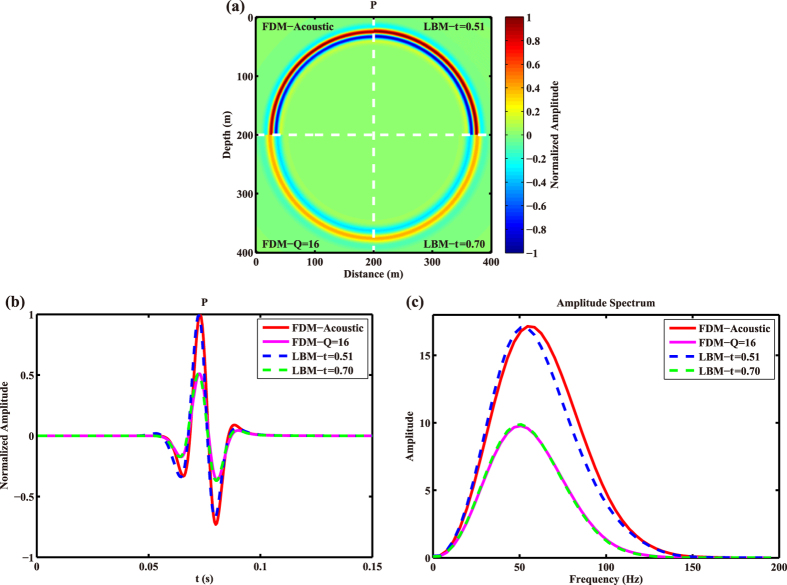
Snapshots (**a**), seismic traces (**b**) and the corresponding amplitude spectra (**c**) obtained by acoustic FDM, viscoacoustic FDM (Q = 16), and LBM with *τ* = 0.51 and *τ* = 0.70.

A parameter which is frequently used to describe the attenuation of media is Q value^34^, which is defined as the ratio of the total energy in a system to the energy lost per cycle. We want to find out the relationship between the relaxation time in LBM and Q. By fixing the Q value in Kelvin-Voigt FDM^35, 36^, then slowly changing the value of *τ*, and comparing the wavefields, seismic traces as well as the amplitude spectra corresponding to the traces calculated by LBM and FDM, we find that the two parameters of Q and *τ* do have a relationship. For example, when the relaxation time is chosen as 0.70, the wavefront calculated by LBM is very similar with that by FDM with Q = 16. Another case is for the relaxation time of *τ* = 0.51, whose wavefield is similar to the result by acoustic FDM, i.e., Q value is very large. The results for the above two cases are shown in Fig. 2a and b, and the corresponding amplitude spectra of the seismic traces are shown in Fig. 2c.

Based on Fig. 2a and b, we can see the snapshot by LBM with the relaxation time of 0.70 is almost the same as the wavefield by FDM with Q = 16, their amplitudes as well as phases are hard to distinguish. On the other hand, when the relaxation time is close to the limiting value of 0.50, the simulation results by LBM can roughly reproduce the wavefield by acoustic FDM. This phenomenon of the two cases is further verified by the amplitude spectra shown in Fig. 2c, in which the curve by LBM with *τ* = 0.70 overlaps that by FDM with Q = 16. Meanwhile, the amplitude spectrum by LBM with *τ* = 0.51 is in close proximity to the case of acoustic wave by FDM. The similarity implies that the relaxation time do have a relationship with the quality factor. To investigate the quantitative relationship between them, we have conducted numerous experiments with different cases of time intervals and dominant frequencies, and found similar sets of Q and *τ* among which six groups of Q and *τ* are listed in Table 1.

**Table 1:** The relaxation time *τ*, and its equivalent quality factor Q for different cases of time interval Δ*t* and dominant frequency *f*_*m*_.

Δ*t*, *f*_*m *_ *τ*	0.505	0.51	0.515	0.52	0.53	0.54	0.55	0.58	0.60	0.65	0.70	0.75	0.80	0.90
0.50 ms, 25 Hz	1200	630	424	318	206	150	122	76	62	42	32	26	22	19
0.50 ms, 50 Hz	600	300	207	158	103	75	59	38	31	21	16	13	10	8
0.50 ms, 75 Hz	410	212	142	106	62	50	39	25	20	14	11	9	7.5	6
0.25 ms, 50 Hz	1273	636	420	318	206	150	122	76	62	42	32	26	22	19
0.50 ms, 50 Hz	600	300	207	158	103	75	59	38	31	21	16	13	10	8
1.00 ms, 50 Hz	305	150	106	79	49	37	29	19	15.5	10.5	8	6.5	5.5	4.5

### Layered model

**Figure 3:**
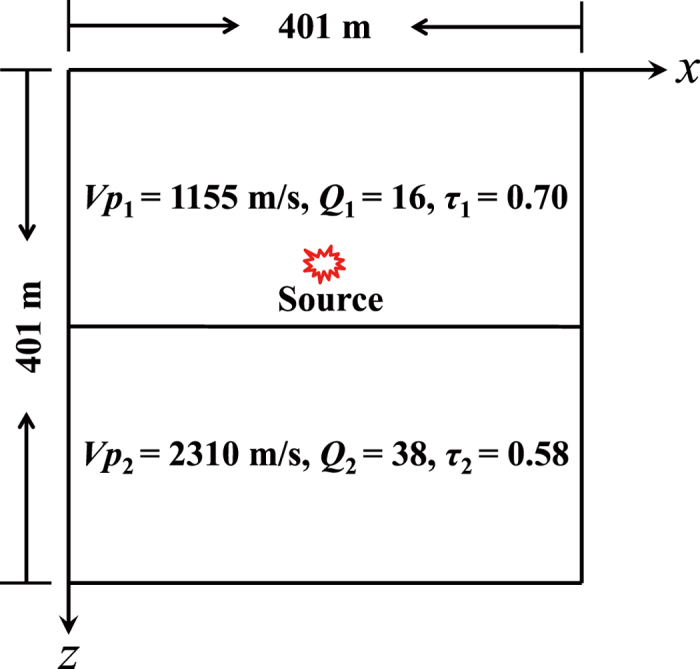
Diagram of the layered model.

**Figure 4:**
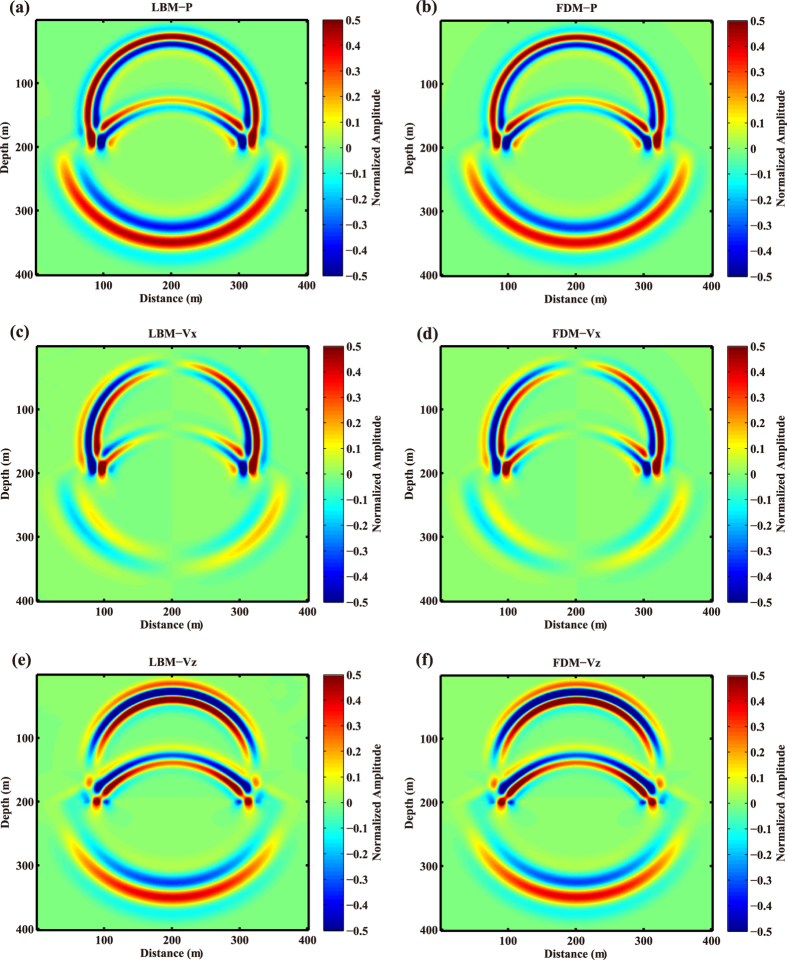
Snapshots of P (**a**) and (**b**), *V*_*x*_ (**c**) and (**d**), *V*_*z*_ (**e**) and (**f**) calculated by LBM (left column) and FDM (right column) for the layered model.

**Figure 5:**
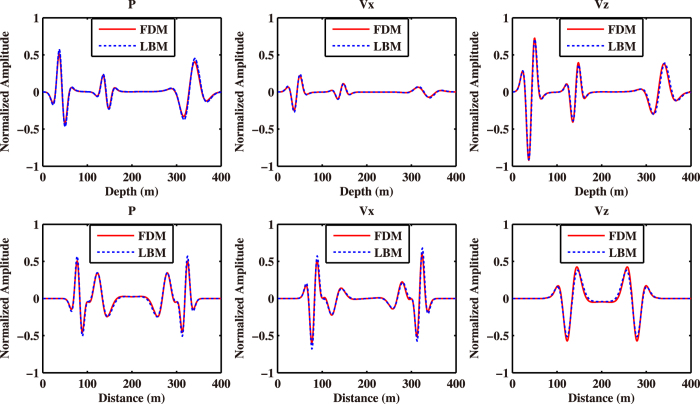
Profiles of wavefields obtained by LBM (dashed line) and FDM (solid line) for the layered model. The left, middle and right columns represent P, *V*_*x*_, and *V*_*z*_, respectively. The top row stands for the wave profiles at *x* = 150 m, and bottom row stands for the wave profiles at *z* = 150 m.

As described in the previous section, we have built a model between the relaxation time and the quality factor. Now, we use a two-layer model with different velocities, Q and relaxation times to validate the relationship. The model is shown in Fig. 3, in which the P-wave velocities of the upper and lower layers are 1,155 m/s and 2,310 m/s, respectively. The discrete grids are 401 × 401, and the spatial intervals along the horizontal and vertical directions are both 1.0 m. The interface is located at 201 m in depth and the source coordinate is (201 m, 151 m). In this experiment, the wavefields by LBM with *τ*_1_ = 0.70, *τ*_2_ = 0.58 and FDM with Q_1_ = 16, Q_2_ = 38 for the upper and lower layers are obtained for comparison. The numerical simulation results are shown in Figs 4 and 5. Fig. 4 depicts the snapshots of P, *V*_*x*_ and *V*_*z*_ obtained by both LBM and FDM. One can see from the figure that the direct waves, reflected waves and transmitted waves calculated by LBM are almost the same as those by FDM. Furthermore, we extract wave profiles at 150 m offset and 250 m depth from the results by both LBM and FDM, and show them in Fig. 5. According to Figs 4 and 5, one can find that the wavefields by LBM coincide with those by FDM.

Of course, to produce the same wavefield by the two schemes of LBM and FDM, any set of relaxation time and quality factor can be found easily through the relationship given by Eq. 1 in the next subsection of “Relationship between Q and *τ*”.

### Relationship between Q and *τ*

**Figure 6:**
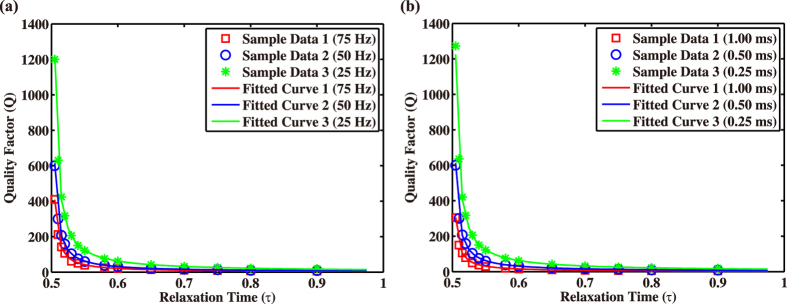
Relationship between quality factor Q and relaxation time *τ* with (**a**) the fixed time interval of 0.5 ms, and (**b**) the fixed dominant frequency of 50 Hz.

Based on the data listed in Table 1, we build a cross-plot of relaxation time and the quality factor in Fig. 6 that the two variables should have a unique relationship. We find the following function to fit it,

1 $$Q=\frac{a\tau +b}{{f}_{m}{\rm{\Delta }}t(\tau -0.5)},$$

where *f*_*m*_ is the dominant frequency of the source, Δ*t* is the time interval used in the simulation, *a* = 0.0192 and *b* = 0.0669 are two constants. The confidence bounds for *a* and *b* are (−0.0426, 0.0810) and (0.0354, 0.0984), respectively. We need emphasize that the Q value in Eq. 1 is the quality factor corresponding to the case when the reference frequency is chosen as the dominant frequency of the seismic source, it is not a frequency dependent value, but Q is a fixed value once we know the dominant frequency of the source.

Fig. 6a shows the case of fixed time interval of 0.5 ms, in which the sample data for the dominant frequencies of 25 Hz, 50 Hz and 75 Hz are shown by red squares, blue circles and green stars, and the corresponding fitting curves are shown by the solid lines, respectively. Fig. 6b shows the case of fixed dominant frequency of 50 Hz, the sample data and the fitting curves for the time intervals of 0.25 ms, 0.50 ms and 1.00 ms are indicated by stars, circles and squares, and the corresponding fitting curves are shown by the solid lines. Obviously, the sample data drawn according to the data in Table 1 fit very well with the solid lines given by Eq. 1. The maximum total relative error between the predicted Q values calculated by the fitting function and the actual Q values is 0.08%. In order to verify the correctness of Eq. 1, numerical experiments of a two-layered model are conducted as shown in the previous subsection of “Layered model”.

## Discussions

Based on the numerical experiments of the homogeneous and layered models, we can see that the lattice Boltzmann equation with one relaxation time can be used to model viscoacoustic wave propagation in viscous media. Therefore, a new scheme different from the traditional FDM is offered to simulate the viscoelastic waves. Since such a novel scheme is not based on the conventional wave equations, vast numerical experiments are carried out to test its correctness and effectiveness. In this process, we acquire different relaxation time in LBM simulation and compare the resultant wavefields with those given by viscoacoustic FDM with different values of Q.

By comparing the wavefields and their amplitude spectra, we find a relationship between the relaxation time and Q as expressed in Eq. 1. We can learn from this equation as well as the curves in Fig. 6 that once the time interval and the dominant frequency are chosen, Q decreases with the relaxation time, and more importantly, when the relaxation time approaches to 0.50, Q reaches infinity, which corresponds to the acoustic wave propagation case. On the other hand, when the relaxation time increases, Q decreases fast and approaches to zero, but could not be equal to zero. A large relaxation time means the resultant LBM wavefields suffer from serious attenuation and dispersion. The results also demonstrate that LBM can be used to capture wave propagating in the earth accompanies with amplitude attenuation and phase dispersion. As some realistic media may contain fluid with different components, which are hard to be depicted with the traditional viscoacoustic models, while LBM scheme is suitable to simulate wave propagation in multiphase media, so it is meaningful to develop such a novel scheme to help us further understanding wave propagation in complex media.

LBM is a discrete method used for describing the wavefield evolution at the micro-level, which is independent of the traditional wave equations. The relationship between the characterization parameter of LBM and Q has been proved. Numerical experiments have demonstrated the fact that LBM is suitable for simulating seismic P-wave in viscous media, and the impact of viscosity on amplitude and phase is discussed. With further development, LBM could be an effective tool for modelling seismic waves in heterogeneous viscoelastic media and can serve as an alternative forward modelling kernel for seismic inversion and migration. Though the above experiments are all conducted in 2D space with the D2Q9 model, the simulation can be easily extended to 3D case with the D3Q19 model. Besides, the work in this paper is focused on the single-relaxation time case, and one can develop the model by adopting the multiple-relaxation time LBM^37^ to depict the viscous effects which are different from the Kelvin-Voigt model, but such work is beyond the scope of this paper.

## Methods

**Figure 7:**
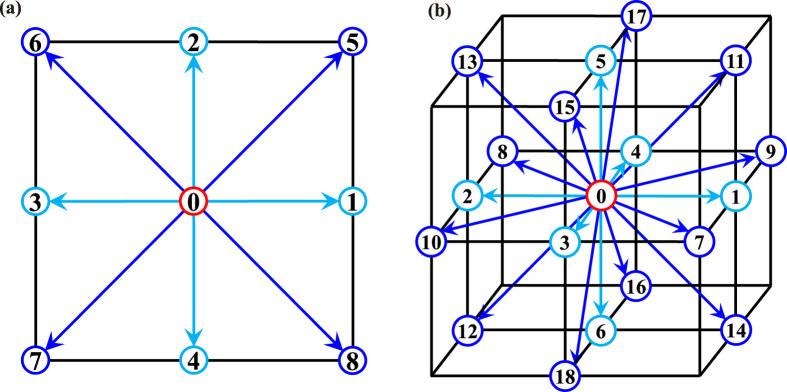
Sketch maps for the LBM discrete models of (**a**) D2Q9, and (**b**) D3Q19.

LBM uses real numbers instead of bits to represent particle distributions, and is an extension of LGA^18^. In LBM, space is discretized by a three-dimensional cubic lattice, time and the velocity discretized as well. The fluid media can be characterized by a single particle velocity distribution function ${f}_{i}({\bf{x}},t)\equiv {f}_{i}({\bf{x}},{{\bf{c}}}_{i},t),i=1,\,2,\,\cdots ,q$, describing the mass density of fluid particles with velocity c_*i*_ at a lattice node **x** and at time *t*. The widely adopted notation for LBM is DdQq, where D stands for space dimension and Q for the number of discrete velocities^20^. The most commonly used models are D2Q7, D2Q9, D3Q15 and D3Q19. Here, some details on D2Q9 (Fig. 7a) and D3Q19 (Fig. 7b) are listed. For the case that the spatial interval Δ*x* and temporal interval Δ*t* are both equal to 1, 9 velocities of D2Q9 are denoted as

2 $${{\bf{c}}}_{i}=n{{\bf{e}}}_{i}=n\{\begin{array}{ll}(\mathrm{0,}\,0) & i=\mathrm{0,}\\ (\pm \mathrm{1,}\,0),(\mathrm{0,}\pm 1) & i=\mathrm{1,}\,\mathrm{2,}\,\mathrm{3,}\,\mathrm{4,}\\ (\pm \mathrm{1,}\pm 1) & i=\mathrm{5,}\,\mathrm{6,}\,\mathrm{7,}\,\mathrm{8,}\end{array}$$

and the corresponding weights are defined by

3 $${w}_{i}=\{\begin{array}{ll}\mathrm{4/9} & \parallel {{\bf{e}}}_{i}\parallel \mathrm{=0,}\\ \mathrm{1/9} & \parallel {{\bf{e}}}_{i}\parallel \mathrm{=1,}\\ \mathrm{1/36} & \parallel {{\bf{e}}}_{i}\parallel =\sqrt{2},\end{array}$$

where *n* is the number of grids particles jumping within the time step of Δ*t*, **e**_*i*_ are the basic lattice vectors. The discrete velocities and weights for D3Q19 are

4 $${{\bf{c}}}_{i}=n{{\bf{e}}}_{i}=n\{\begin{array}{cc}(0,\,0,\,0) & i=0,\\ (\pm 1,0,0),(0,\pm 1,\,0),(0,\,0,\pm 1) & i=1,\cdots ,\,6,\\ (\pm 1,\pm 1,\,0),(\pm 1,0,\pm 1),(0,\pm 1,\pm 1) & i=7,\,\cdots ,\,18,\end{array}$$

5 $${w}_{i}=\{\begin{array}{ll}\mathrm{1/3} & \parallel {{\bf{e}}}_{i}\parallel \mathrm{=0,}\\ \mathrm{1/18} & \parallel {{\bf{e}}}_{i}\parallel \mathrm{=1,}\\ \mathrm{1/36} & \parallel {{\bf{e}}}_{i}\parallel =\sqrt{2},\end{array}$$

The simulation of wave propagation using LBM mainly contains two processes. The first one is the propagation of fluid particles to adjacent lattice sites (the streaming step), and the second one is collisions among particles when they reach a site at the same time (the collision step). The streaming step is

6a $${f}_{i}({\bf{x}}+{{\bf{c}}}_{i},t+1)={f^{\prime} }_{i}({\bf{x}},t),$$

and the collision step is

6b $${f^{\prime} }_{i}({\bf{x}},t)={f}_{i}({\bf{x}},t)-\frac{1}{\tau }[{f}_{i}({\bf{x}},t)-{f}_{i}^{(eq)}({\bf{x}},t)],$$

Combining these two steps, the lattice Boltzmann equation^20^, which controls the evolution of the particle distribution function, can be denoted as

7 $${f}_{i}({\bf{x}}+{{\bf{c}}}_{i},t+1)={f}_{i}({\bf{x}},t)-\frac{1}{\tau }[{f}_{i}({\bf{x}},t)-{f}_{i}^{(eq)}({\bf{x}},t)],$$

where ${f}_{i}^{(eq)}({\bf{x}},t)$ is the equilibrium distribution and *τ* is a relaxation time^32^, which is related to the viscosity of the media as $v={c}_{s}^{2}(\tau -0.5)$. For the DdQq models in Cartesian coordinate system, the equilibrium distribution^20^ can be expressed as

8 $${f}_{i}^{(eq)}={w}_{i}\rho [1+\frac{1}{{c}_{s}^{2}}({{\bf{c}}}_{i}\cdot {\bf{u}})+\frac{1}{2{c}_{s}^{4}}{({{\bf{c}}}_{i}\cdot {\bf{u}})}^{2}-\frac{1}{2{c}_{s}^{2}}|{\bf{u}}{|}^{2}],$$

where *c*_*s*_ is the lattice speed of sound and equals to $\mathrm{1/}\sqrt{3}$ for D2Q9 and D3Q19, *w*_*i*_ is the weighting factor which ensures that the lattices satisfy certain symmetrical properties necessary for isotropic behavior, *ρ* and **u** are the fluid density and velocity at the macroscopic level, respectively. *ρ* and **u** can be calculated by

9a $$\rho =\sum _{i}{f}_{i},$$

9b $${\bf{u}}=\frac{\sum _{i}{f}_{i}{{\bf{c}}}_{i}}{n\rho },$$

Eqs 9a and 9b links the microscopic parameters of LBM with the wavefield variables at the macroscopic level. As the viscoacoustic wave equation of the Kelvin-Voigt model can be deduced from the original Navier-Stokes equation^34^, and LBM is an effective discrete scheme of the Navier-Stokes equation^20^, so it is reasonable to match the wavefields by LBM with those by FDM.
